# Oncofertility Knowledge and Communication: Comparison Between Medical and Surgical Oncologists and Breast Cancer Patients in Academic Chinese Centers

**DOI:** 10.3389/fsurg.2021.681614

**Published:** 2021-09-07

**Authors:** Ewelina Biskup, Zhaochen Xin, Rui Li, John P. Zucal, Yao Lu, Yun Sun, Leslie Coker Appiah, Steven R. Lindheim, Hongwei Zhang

**Affiliations:** ^1^Department of Medical Oncology, Renji Hospital, School of Medicine, Shanghai Jiao Tong University, Shanghai, China; ^2^Department of Basic and Clinical Medicine, Shanghai University of Medicine and Health Sciences, Shanghai, China; ^3^International Center for Multimorbidity and Complexity in Medicine (ICMC), Universität Zürich, Zürich, Switzerland; ^4^Department of General Surgery, Zhongshan Hospital, Fudan University, Shanghai, China; ^5^Department of Public Health Sciences, University of Rochester School of Medicine and Dentistry, Rochester, NY, United States; ^6^Wright State University Boonshoft School of Medicine, Dayton, OH, United States; ^7^Center for Reproductive Medicine, School of Medicine, Renji Hospital, Shanghai Jiao Tong University, Shanghai, China; ^8^Shanghai Key Laboratory for Assisted Reproduction and Reproductive Genetics, Shanghai, China; ^9^Department of Obstetrics and Gynecology, The University of Colorado, Denver, CO, United States

**Keywords:** oncofertility, fertility preservation, breast cancer, breast cancer survivors, oncology, oncologist

## Abstract

**Background:** As cancer has become a major public health issue in China, fertility preservation remains limited despite the wide application of Assisted Reproductive Technology (ART) throughout the country.

**Objective:** This study aimed to identify gaps in knowledge and communication as well as referrals in the previous year regarding oncofertility among medical and surgical oncologists and breast cancer patients (BCPs) in Chinese academic settings to target areas of needed improvement.

**Materials and Methods:** A WeChat online questionnaire was designed, distributed, and compared between medical and surgical oncology specialists and reproductive age BCPs in academic teaching settings in Shanghai.

**Results:** Sixty-one medical and surgical oncologists and 125 BCPs responded to the survey. 63.3% of oncologists were familiar with the term “oncofertility” compared to 25.6% of BCPs (*p* < 0.001). Oncologists were more likely to correctly know the costs associated with treatment (59.0 vs. 32.0%, *p* < 0.001); patient did not have to be married to undergo oncofertility treatment (50.8 vs. 24.8%, *p* < 0.001). Both oncologists and BCPs were similarly unlikely to know when patients could utilize cryopreserved tissue in the future (37.7 vs. 22.2%, *p* = 0.056). While oncologists reported they discussed all oncofertility options (41.0%) and offered psychological counseling (98.4%), significantly fewer BCPs reported receiving information on all options and offered counseling (3.2%, *p* < 0.001 and 85.6%, *p* < 0.01). Knowledge of oncofertility was the most important predictor for providing and receiving counseling from oncologists [OR = 6.44 (95% CI = 1.59–26.1, *p* = 0.009] and BCPs (OR = 3.73 95% CI: = 1.36–10.2, *p* = 0.011). Overall, 57.4% of oncologists referred <10 patients and none referred more than 25 patients in the past year.

**Conclusion:** Data suggests a significant knowledge gap and ineffective communication/comprehension exists between academic Chinese oncologists and BCPs. Continued education and raised awareness are needed to optimize utilization of oncofertility services in China.

## Introduction

Cancer incidence and mortality continue to increase world-wide, despite rather stable rates over the last decade in the Western countries (1). This positions cancer as one of the top global burden diseases (2). China takes a special position within the international oncological arena due to the large population and severe regional disparities in cancer epidemiology. The reported incidence of all cancers by the China National Cancer Center remains on the rise, with 3,929,000 in 2015 and 4,285,033 cases in 2018 (3). While cancer is mostly a disease of the elderly, there remains a large number of cancer types in young adults and adolescents, e.g., breast and colon cancer which includes nearly 400,000 reproductive age adults and 23,000 pre- and post-adolescents (4). Fortunately, mortality rates both globally and in China have decreased due to advancements in treatment regimens (1, 5), thus, leading to a rise in the number of cancer survivors in pre- and reproductive age (6, 7).

Cancer treatments have the potential to have highly detrimental effects on gamete function and many adolescent and young adult (AYA) cancer survivors face the prospect of infertility caused by the disease process and/or the cancer treatment itself (8–10). In survey based studies, over half (51.7%) of young cancer survivors describe parenthood as the “most important” issue in their life with many wishing to use their own oocytes and the risk of treatment-related infertility even affecting their decision making about undergoing recommended cancer treatments (11–13). Moreover, even for those who initially stated that fertility preservation was not that important to them, (14) has reported that the issue of fertility becomes increasingly important over time (15).

Novel approaches in preventative therapy and preserving the potential for future fertility for cancer patients have become standard including gamete, embryo, and ovarian tissue cryopreservation (OTC) (12, 16). As such, numerous international fertility preservation and restoration guidelines have been published by American Society of Clinical Oncology (ASCO) (17–19), American Society for Reproductive Medicine (ASRM) (20, 21), European Society for Medical Oncology (21–23), American Oncofertility Consortium (OC) (24, 25), International Society for Fertility Preservation (ISFP) (19, 26–28), National Comprehensive Cancer Network (NCCN), American Academy of Pediatrics (AAP), Association of Pediatric Hematology/Oncology Nurses (APHON) (18), and the German Fertility Preservation Network (FertiPROTEKT) (29) endorsing the requirements that all AYA are to be counseled about the gonadotoxic risk of anticancer therapy, with timely referrals to reproductive specialists in order to provide information, treatment options and follow up.

Despite the interest in parenthood expressed by many cancer patients, the number of patients who access fertility preservation remains relatively low (30). Patients' lack of awareness of treatment-related infertility, together with the time pressures and conflicting priorities of physicians are among the many factors which may hinder adequate oncologist-patient fertility discussions and timely referrals (31). In China, as cancer has become a major public health issue, fertility preservation remains limited despite the wide application of Assisted Reproductive Technology (ART) throughout the country for more than 30 years. Meanwhile, the services for ART are available for all oncology patients in China, albeit not fully covered by the national insurance. Females undergoing oncofertility treatment can in principle access their gametes at any point of time in the future (subject to specific limitations in terms of storage duration). Being married is not a requested in order to preserve fertility in cancer patients (as opposed to social freezing). Lack of oncofertility integration into the Chinese medical field, failure to use ovarian protective strategies such as gonadotropin-releasing hormone agonist treatment in certain populations and regulations that restrict donor oocytes and gestational surrogacy have been cited as reasons for its restricted use (20, 32, 33).

Marginal data exist on fertility preservation for cancer patients in China. Biskup et al. (19) recently reported the number of oncofertility cases performed in the past 5 years at one level 3 teaching hospital, which are the only facilities allowed to provide ART services in China, included just 270 semen and 14 cases of ovarian tissue cryopreservation, while oocyte and embryo cryopreservation were not performed. In comparison, USA long term storage facilities including California Cryobank and Fairfax Cryobank, Inc., performed 1,550 and 768 cases of semen cryopreservation for oncofertility, respectively, and REPROTECH LIMITED had requests for banking that increased over 200%^.^ For females, between 2007 and 2017, 420 underwent OTC at National Physicians Cooperative (NPC) member institutions of the OC^19^.

More recently, the impetus to overcome these barriers and to enhance awareness of fertility preservation options for oncofertility patients in China has moved forward with the establishment of evolving guidelines and regulations. In 2017, the Chinese Society of Oncofertility was created, yet, as of 2018 there were no ART programs in Shanghai that were part of the OC-NPC. To further highlight the limited use of these services among Chinese reproductive health professionals, Ju et al. reported on the overall the lack of oncofertility knowledge in Fujan, a province in the Southeast part of China, where there is a relatively high incidence in breast cancer in young females (20). The authors highlighted the need for continued oncofertility education and training. Biskup et al. (19) additionally suggested that oncofertility utilization may be further limited due to mistaken provider beliefs and complex social and cultural attitudes among patients regarding fertility in general and fertility preservation.

Given that female fertility preservation is more complex than male preservation, and the high volume work-load of Chinese health-care providers, we objectively sought to assess knowledge, attitudes, and communication regarding oncofertility services among academic medical and surgical oncologist compared to BCPs. The aim was to identify gaps in knowledge and communication as a strategy to target areas of improvement.

## Materials and Methods

### Ethical Approval

Medical and surgical oncologists and BCP from five academic hospitals including Zhongshan Hospital, Fudan University; Changhai Hospital, Second Military Medical University; Medical College of Shanghai Jiaotong University; International Peace Maternal and Child Health Hospital, Medical College of Shanghai Jiaotong University; and Obstetrics and Gynecology Hospital, Fudan University, were surveyed between June 2019 and August 2019.

### Study Protocol

All attending and resident physicians (*n* = 77) from each of the five medical and surgical oncology departments were invited to participate (80% response rate) in a 30-question online survey while BCPs were asked to complete a 20-question online survey. Using a Wenjuanxing (WJX) survey system, a link with the survey questions was created and distributed *via* the WeChat application directly to the respective departmental physicians. BCPs received the link to their survey either directly from their physician/nurse, and were asked to share the link among other BCPs. Surveys responses were automatically stored in an electronic, exportable database of WJX. Confidentiality was protected with unique identification numbers that were used on all data collection forms and when performing statistical analyses. Each participant read and signed an informed consent prior to taking the survey. The survey was anonymous and confidentiality was protected with unique identification numbers when performing statistical analysis.

### Variables Studied

The surveys (attached in Appendices A, B) were translated from English to Mandarin and consisted of five domains for HCPs including demographics, knowledge, services offered, attitudes, and utilization; for BCPs, domains included: demographics, knowledge, and services discussed with oncology provider.

### Statistical Analyses

Descriptive statistics were performed using means with standard deviations for continuous variables, and frequencies and percentages for categorical variables. Differences between medical and surgical oncologists, as well as between oncologists and patients, were compared using the 2-sample *t*-test, Chi-square test, or Fisher's exact test. A sum knowledge score was constructed based on the number of correctly answered questions regarding oncofertility knowledge (e.g., correctly knowing the estimated cost of oocyte/embryo cryopreservation). Univariate logistic regression models examined associations between each physician and patient characteristics and “knowing what oncofertility was” and “discussing oncofertility,” respectively. Variables significant at an alpha level of 0.2 were then entered into multiple logistic regression models. Odds ratios (ORs) with 95% confidence intervals (95% CIs) were used to describe the magnitude of associations. All statistical tests were two-sided, and *p* < 0.05 were considered statistically significant. Data management and statistical analyses were conducted in SAS version 9.4 (SAS Inc., Cary, NC).

## Results

### HCP and Patient Demographics

Medical and surgical oncologists and patient characteristics are displayed in Tables 1, 2. A total of 61 of oncologists responded including 28 medical oncologists and 33 surgical oncologists. The mean age of responding physicians were 35.7 years (range, 27–55 years); most were in practice for 11–20 years (36.1%); and 60.7% were male. Surgical oncologists were older and in practice longer than medical oncologists. One-hundred-twenty-five BCP respondents completed the survey with a mean age of 40.9 years, (range 23–49 years); 44.0% lived in Shanghai and 12.0% were from rural areas; 52.8% had a university education; 78.4% were married with children. Breast cancer stage at initial time of diagnosis included 16% Stage 1; 38.4% Stage II; 14.4% Stage III; 5.4 % Stage IV; and 24.8% did not know.

**Table 1:** Physician demographics (*n* = 61).

**Characteristics**	**All oncologists (61)**	**Medical oncologists (28)**	**Surgical oncologists (33)**	***p*-value**
**Physician age (years)**				
Mean ± SD^a^	35.7 ± 6.0	33.7 ± 6.3	37.4 ± 5.2	0.014
25–30 years (residents)	12 (19.7)^*^	10 (35.7)	2 (6.1)	0.004^b^
31–50 years (attending physicians)	48 (78.7)	17 (60.7)	31 (93.9)	
51 years or above (attending physicians)	1 (1.6)	1 (3.6)	0 (0)	
**Experience of physician**				
<5 years	15 (24.6)	13 (46.4)	2 (6.1)	
5–10 years	20 (32.8)	8 (28.6)	12 (36.4)	0.002^b^
11–20 years	22 (36.1)	6 (21.4)	16 (48.5)	
>20 years	4 (6.6)	1 (3.6)	3 (9.1)	
**Physician sex**				
Male	37 (60.7)	14 (50.0)	23 (69.7)	0.117
Female	24 (39.3)	14 (50.0)	10 (30.3)	

a *Standard deviation*;

b *Fisher's exact test*.

* *n (%)*.

**Table 2:** Patient demographics (*n* = 125).

**Characteristics**	***n* (%)**
**Patient age (years)**	
Mean ± SD^a^	40.9 ± 5.0
<40 years	42 (33.6)
40–49 years	83 (66.4)
**Where they lived**	
Shanghai	55 (44.0)
Outside Shanghai >1 million inhabitants	34 (27.2)
Outside Shanghai <1 million inhabitants (Rural Area)	21 (16.8)
	15 (12.0)
**Education**	
Primary School	10 (8.0)
High School	43 (34.4)
University	66 (52.8)
Other	6 (4.8)
**Marital/family status**	
Married with children	98 (78.4)
Married without children	8 (6.4)
Single	7 (5.6)
Divorced with children	10 (8.0)
Boyfriend and no children	2 (1.6)
**Stage of breast cancer**	
I (a-b-or c)	20 (16.0)
II (a or b)	48 (38.4)
III (a-b-or c)	18 (14.4)
IV (palliative)	7 (5.6)
Paget disease	1 (0.8)
Did not know	31 (24.8)
**Type of therapy**	
Chemotherapy only	34 (27.2)
Radiotherapy only	4 (3.2)
Endocrine therapy only	6 (4.8)
Immunotherapy only	2 (1.6)
Combination therapy	77 (61.6)

a *±Standard deviation*.

### Oncofertility Knowledge

**Figure 1:**
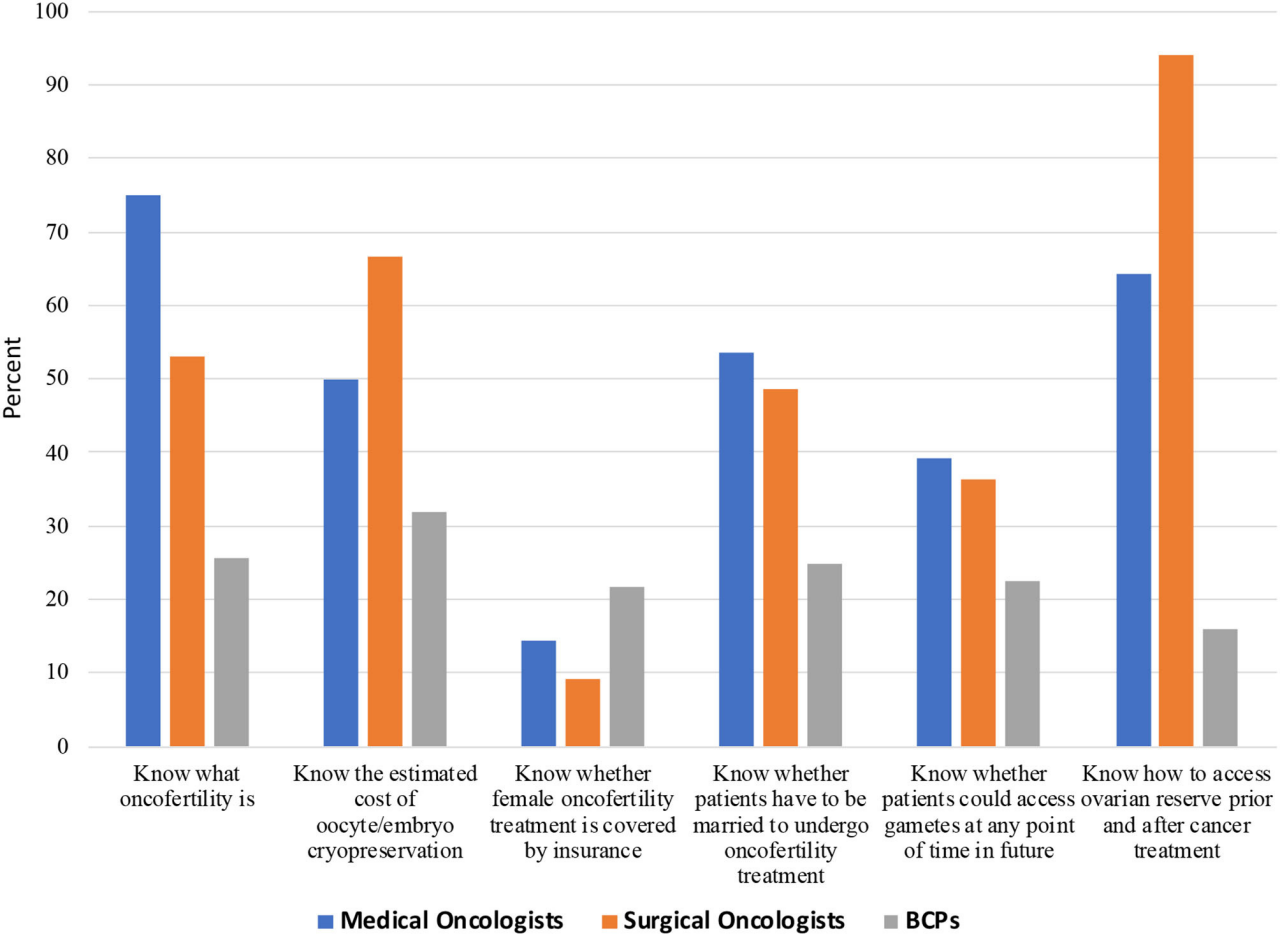
Knowledge of oncofertility among Chinese medical oncologists, surgical oncologists, and breast cancer patients (BCPs).

As depicted in Table 3 and Figure 1, oncologists, overall, knew “what oncofertility was” (63.3%); correctly knew the costs associated with oocyte/embryo cryopreservation (59.0%), the marital status requirements (50.8%), and to assess ovarian reserve with serum cycle day 2 or 3 FSH or AMH (80.3%). However, the majority incorrectly knew or did not know whether oncofertility treatments were covered by insurance (88.5%); and whether those undergoing fertility preservation could access gametes anytime in the future (i.e., required a partner by marriage) (62.3%). While medical oncologists tended to know “what oncofertilty was” (75.0 vs. 53.1%, p-NS), they were less likely to know how to test for ovarian reserve (64.3 vs. 93.9%, *p* = 0.009). Compared to oncologists, BCPs were less likely to know “what oncofertility was” (25.6%, *p* < 0.001); costs associated with treatment (32.0% [59.2% did not know], *p* < 0.001); marital status requirements (24.8%, *p* = 0.001); that they could access their cryopreserved tissue upon marriage (22.4%, p-NS); and know how to test for ovarian reserve (16.0%, *p* < 0.001). Compared to oncologists, they were more likely to know whether oncofertility treatment was covered by insurance (21.6 vs. 11.5%, *p* < 0.001).

**Table 3:** Medical and surgical oncologists and breast cancer patients' knowledge related to oncofertility services.

**Knew the following**	**Medical oncologists (28)**	**Surgical oncologists (33)**	***p*-value**	**All oncologists (61)**	**BCPs (125)**	***p*-value**
**What oncofertility was**						
Yes	21 (75.0)	17 (53.1)^a^	0.079	38 (63.3)^a^	32 (25.6)	<0.001
No	7 (25.0)	15 (46.9)		22 (36.7)	93 (74.4)	
**Estimated cost of oocyte/embryo cryopreservation**						
Correct	14 (50.0)	22 (66.7)	0.187	36 (59.0)	40 (32.0)	<0.001
Incorrect	14 (50.0)	11 (33.3)		25 (41.0)	11 (8.8)	
Did not know	0 (0)	0 (0)		0 (0%)	74 (59.2)	
**Female oncofertility treatment was covered by insurance**						
Correctly knew	4 (14.3)	3 (9.1)	0.475^b^	7 (11.5)	27 (21.6)	<0.001
Incorrectly knew	0 (0)	0 (0)		0 (0)	26 (20.8)	
Did not know	15 (53.6)	23 (69.7)		38 (62.3)	72 (57.6)	
Did not answer	9 (32.1)	7 (21.2)		16 (26.2)	0 (0)	
**Patient does have to be married to undergo oncofertility treatment**						
Correctly knew	15 (53.6)	16 (48.5)	0.87	31 (50.8)	31 (24.8)	0.001
Incorrectly knew	2 (7.1)	4 (12.1)		6 (9.8)	31 (24.8)	
Did not know	11 (39.3)	13 (39.4)		24 (39.3)	63 (50.4)	
**Could access gametes at any point of time in future**						
Correctly knew	11 (39.3)	12 (36.4)	0.560b	23 (37.7)	28 (22.4)	0.056
Incorrectly knew	4 (14.3)	2 (6.1)		6 (9.8)	23 (18.4)	
Did not know	13 (46.4)	19 (57.6)		32 (52.5)	74 (59.2)	
**How to assess ovarian reserve prior and after cancer treatment**						
Correct	18 (64.3)	31 (93.9)	0.009^b^	49 (80.3)	20 (16.0)	<0.001^b^
Would not do	2 (7.1)	0 (0)		2 (3.3)	–	
Did not know	8 (28.6)	2 (6.1)		10 (16.4)	105 (84.0)	

a *One surgical oncologist did not answer this question*;

b *Fisher's exact test*.

### Attitudes, Services Offered, and Utilization

**Figure 2:**
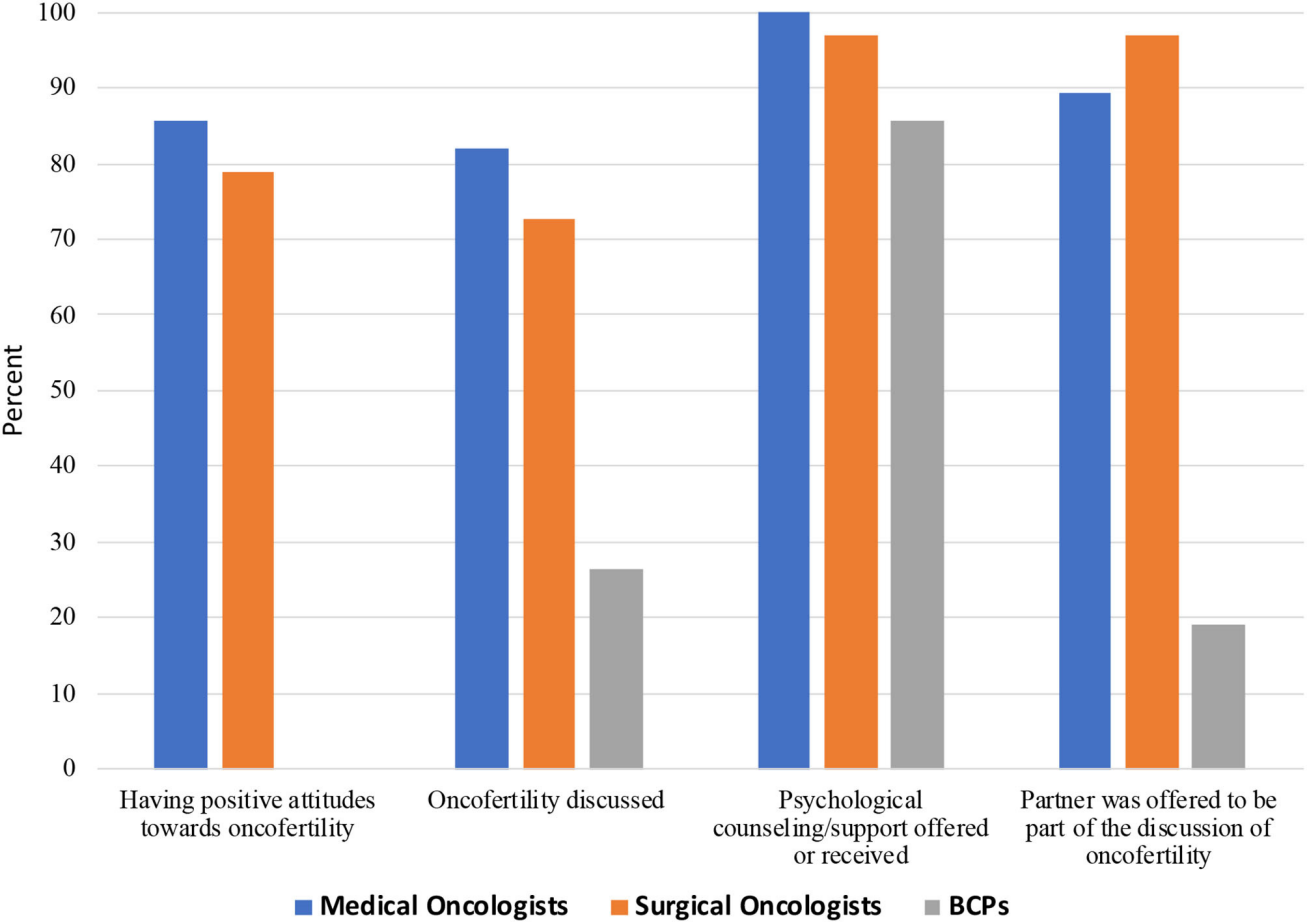
Attitudes and discussion of oncofertility treatment among Chinese medical oncologists, surgical oncologists, and breast cancer patients (BCPs).

As shown in Table 4 and Figure 2, overall, 82.0% of oncologists had a positive attitude toward oncofertility treatment, but were less likely to discuss these options with pre-adolescent girls than adolescent and reproductive age women (57.4 vs. 82 and 77.1%, *p* < 0.001). Significantly more oncologists stated they provided information regarding oncofertility; offered psychological support/counseling; and allowed the partner (if applicable) to be part of the discussion (77.1, 98.4, and 93.4%, respectively) compared to 26.4; 85.6, and 19.2% of BCPs. No differences were noted between medical and surgical oncologists.

**Table 4:** Treatment-services discussed and attitudes related to oncofertility services.

	**Medical oncologists (28)**	**Surgical oncologists (33)**	***p*-value**	**All oncologists (61)**	**BCPs (125)**	***p*-value**
**Positive attitudes toward oncofertility**						
Yes	24 (85.7)^*^	26 (78.8)	0.41	50 (82.0)	–	–
No	1 (3.6)	1 (3.0)		2 (3.3)		
Did not know	3 (10.7)	6 (18.2)		9 (14.7)		
**Oncofertility discussed with provider/reproductive age patient**						
Yes	23 (82.1)	24 (72.7)	0.384	47 (77.1)	33 (26.4)	<0.001
No	5 (17.9)	9 (27.3)		14 (22.9)	92 (73.6)	
**Specific oncofertility options discussed**						
Egg Banking	23 (82.1)	28 (84.8)	1	51 (83.6)	67 (53.6)	<0.001
Embryo Banking	20 (71.4)	27 (81.8)	0.336	47 (77.0)	36 (28.8)	<0.001
OTC^a^	18 (64.3)	25 (75.8)	0.328	43 (70.5)	16 (12.8)	<0.001
GnRH-a therapy	18 (64.3)	17 (51.5)	0.315	35 (57.4)	7 (5.6)	<0.001
All options	12 (42.9)	13 (39.4)	0.784	25 (41.0)	4 (3.2)	<0.001
**Oncofertility discussed by provider to adolescent patients**						
Yes	25 (89.3)	25 (75.8)	0.171	50 (82.0)	–	–
No	3 (10.7)	8 (24.2)		11 (18.0)		
**Oncofertility discussed by provider to pre-adolescent patients**						
Yes	17 (60.7)	18 (54.5)	0.627	35 (57.4)	–	–
No	11 (39.3)	15 (45.5)		26 (42.6)		
**Psychological counseling/support offered**						
Yes	28 (100)	32 (97.0)	1.000b	60 (98.4)	107 (85.6)	0.007
No	0 (0)	1 (3.3)		1 (1.6)	18 (14.4)	
**Partner was offered to be part of the discussion of oncofertility**						
Yes	25 (89.3)	32 (97.0)	0.325^b^	57 (93.4)	24 (19.2)	
No	3 (10.7)	1 (3.0)		4 (6.6)	91 (72.8)	<0.001
No partner				–	10 (8)	

a *Ovarian Tissue Cryopreservation*;

b *Fisher's exact test*.

* *n (%)*.

**Figure 3:**
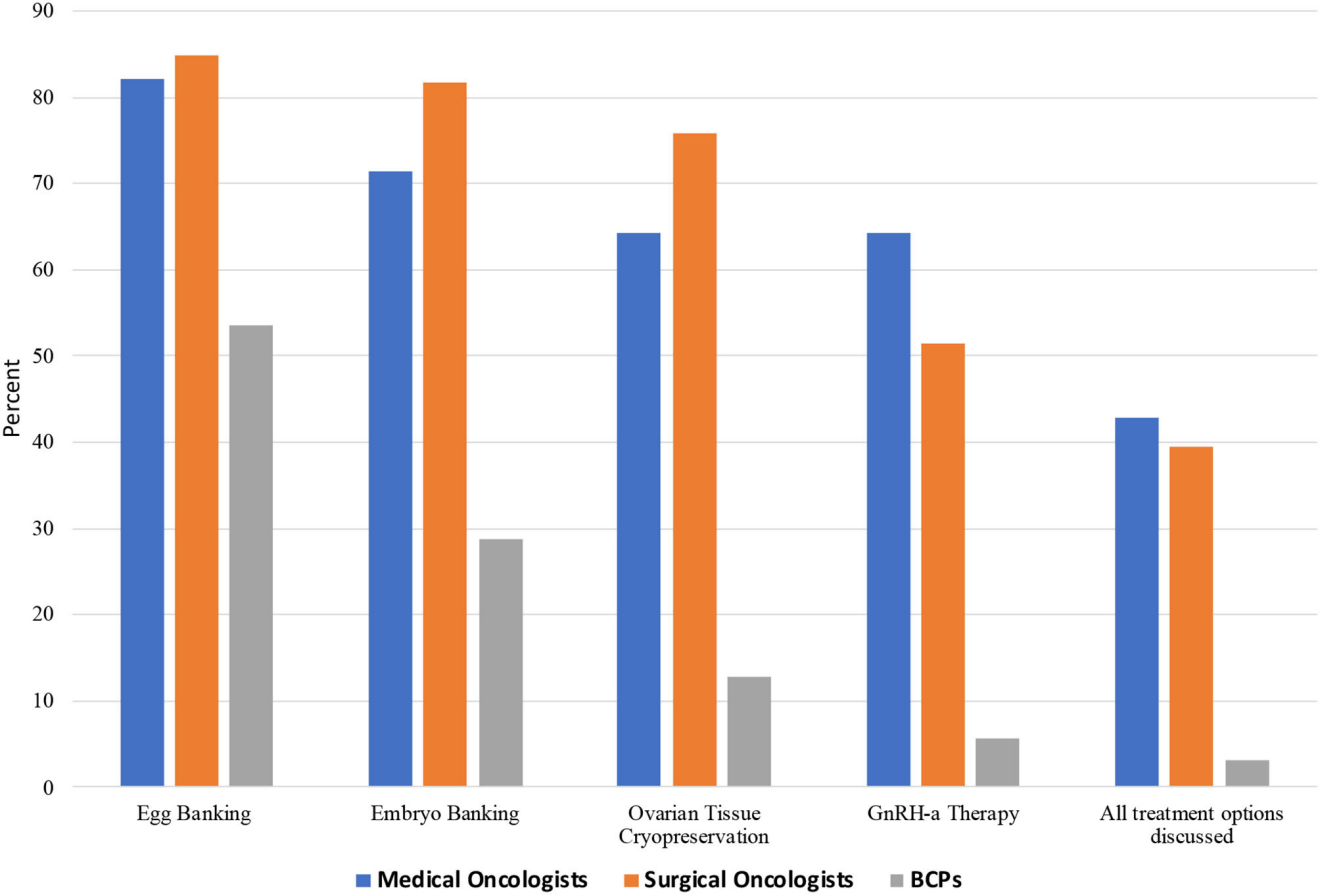
Specific oncofertility options discussed among Chinese medical oncologists, surgical oncologists, and breast cancer patients (BCPs).

Over the previous year, 64.3% of oncologists reported referring candidates for oocyte-embryo banking <10 times; 1.9% referred 10–25 times and 1.9%-more than 25 times, while 32.6% did not recall the number of referrals. Reasons given by the oncologists as to why BCPs were not utilizing oncofertility services included: patient's personal wishes (39.3% [24]), financial (39.3% [24]), unaware of options (70.5% [43]), cultural barriers (44.2% [27]), and did not know (4.9% [3]), as shown in Figure 3.

### Factors Associated With Knowledge (Awareness) and Service Offered (or Received) of Oncofertility

Table 5 shows factors associated with “knowing what oncofertility was” among oncologists. Female oncologists, compared to male, and medical oncologists, compared to surgical oncologists, were more likely to know what oncofertility was in both univariate and multiple logistic regression models, although the differences were not significant.

**Table 5:** Factors associated with “knowing what oncofertility was” among oncologists (*n* = 60).

	**Univariate logistic regression**		**Multiple logistic regression^a^**	
	**OR (95% CI)**	***p*-value**	**AOR (95% CI)**	***p*-value**
Age (years)	0.99 (0.90, 1.08)	0.755		
Sex: Male vs. Female	0.42 (0.13, 1.30)	0.13	0.48 (0.15, 1.53)	0.213
Specialty: medical vs. surgical	2.65 (0.88, 7.97)	0.083	2.37 (0.77, 7.29)	0.133
**Years of practice**				
<5 years	Ref			
5–10 years	2.00 (0.47, 8.49)	0.41		
>10 years	0.85 (0.23, 3.11)			

*OR, odds ratio; 95% CI, 95% confidence interval; AOR, adjusted odds ratio*.

a *Only variables with p < 0.2 in the univariate logistic regression models were entered into the multiple logistic regression model*.

Table 6 shows factors associated with “discussing oncofertility with pre-adolescent, adolescent patients and reproductive-aged patients” among oncologists. In univariate models, “knowing what oncofertility was” was associated with 7.08 times the odds (95% CI = 1.87, 26.9) of discussing oncofertility with patients, with each 1-score increase in oncofertility knowledge associated with 81% greater odds of discussion (95% CI = −9%, 261%). A positive attitude toward oncofertility was associated with 3.8 times the odds of discussion (95% CI = 0.95, 15.2). When entered into the multiple logistic regression model, effect sizes attenuated but the direction of associations persisted.

**Table 6:** Factors associated with “discussing oncofertility” with reproductive-aged patients among oncologists (*n* = 61).

	**Univariate logistic regression**		**Multiple logistic regression^a^**	
	**OR (95% CI)**	***p*-value**	**AOR (95% CI)**	***p*-value**
Age (years)	0.98 (0.89, 1.09)	0.744		
Gender: Male vs. female	1.76 (0.53, 5.89)	0.355		
Specialty: Medical vs. surgical	1.72 (0.50, 5.92)	0.387		
**Years of practice**				
<5 years	Ref	0.818		
5–10 years	1.00 (0.19, 5.33)			
>10 years	0.68 (0.15, 3.15)			
“Knowing what oncofertility was”: Yes vs. No	7.08 (1.87, 26.9)	0.004	6.44 (1.59, 26.1)	0.009
Knowledge of oncofertility (for each 1-point increase)	1.81 (0.91, 3.61)	0.091	1.75 (0.84, 3.68)	0.138
Attitude toward oncofertility: Positive vs. Negative	3.80 (0.95, 15.2)	0.06	2.19 (0.47, 10.1)	0.317

*OR, odds ratio; 95% CI, 95% confidence interval; AOR, adjusted odds ratio*.

a *Only variables with p < 0.2 in the univariate logistic regression models were entered into the multiple logistic regression model*.

Table 7 shows factors associated with “knowing what oncofertility was” among BCPs. Univariate models suggested that higher education levels and lower stage of breast cancer were associated with greater likelihood of “knowing what oncofertility was”. Although not statistically significant, patients of younger age, living in Shanghai, and without children tend to be more aware of oncofertility. In multiple logistic regression, only the stage of breast cancer was marginally associated with awareness (*p* = 0.058).

**Table 7:** Factors associated with knowing “what oncofertility was” among breast cancer patients (*n* = 125).

	**Univariate logistic regression (** ***n*** **=** **125)^a^**		**Multiple logistic regression (** ***n*** **=** **119)^b^**	
	**OR (95% CI)**	***p*-value**	**AOR (95% CI)**	***p*-value**
Age (years): <40 vs. 40–49	1.51 (0.66, 3.47)	0.331		
**Where they lived**				
Shanghai	Ref 0.58 (0.21, 1.59)	0.676		
Outside Shanghai ≥1 million inhabitants	0.70 (0.22, 2.22)			
Outside Shanghai <1 million inhabitants Rural Area	0.56 (0.14, 2.24)			
**Education**				
Primary School	2.06 (0.23, 18.6)	0.17	Ref	0.324
High School	4.20 (0.50, 35.3)		1.69 (0.17, 16.5)	
University/Other	1.72 (0.58, 5.11)		3.11 (0.34, 28.1)	
Family Status: Without children vs. With children	1.72 (0.58, 5.11)	0.328		
**Stage of breast cancer**				
I/II (early stage)	0.72 (0.27, 1.96)	0.035	0.77 (0.28, 2.14)	0.058
III/IV (late stage)	0.13 (0.03, 0.62)		0.08 (0.01, 0.64)	
Did not know				
**Type of therapy**				
Chemotherapy only	Ref	0.446		
Chemotherapy not used	0.91 (0.23, 3.57)			
Combination of chemotherapy and others	1.65 (0.62, 4.39)			

*OR, odds ratio; 95% CI, 95% confidence interval; AOR, adjusted odds ratio*.

a *Six patients reported “other” for their education and were excluded from the analysis*.

b *Only variables with p < 0.2 in the univariate logistic regression models were entered into the multiple logistic regression model*.

Table 8 shows factors associated with discussing oncofertility with oncologists among BCPs. Univariate models indicated that patients of younger age, living in Shanghai, of more education, without children, with lower stage of breast cancer, knowing what oncofertility was and having more knowledge of oncofertility, were more likely to discuss oncofertility with their oncologists. Multiple logistic regression indicated that knowing what oncofertility was (OR = 3.73, 95% CI = 1.36, 10.2) and greater knowledge of oncofertility (for 1-score increase, OR = 1.69, 95% CI = 1.14, 2.51), were associated with greater odds of discussing oncofertility with oncologists.

**Table 8:** Factors associated with receiving oncofertility discussion from their oncologist among breast cancer patients (*n* = 125).

	**Univariate logistic regression (** ***n*** **=** **125)^a^**		**Multiple logistic regression (** ***n*** **=** **119)^b^**	
	**OR (95% CI)**	***p*-value**	**AOR (95% CI)**	***p*-value**
Age (years): <40 vs. 40–49	2.01 (0.89, 4.55)	0.096	1.58 (0.57, 4.41)	0.381
**Where they lived**
Shanghai	0.74 (0.29, 1.91)	0.32		
Outside Shanghai ≥1 million inhabitants	0.64 (0.20, 2.03)			
Outside Shanghai <1 million inhabitants (Rural Area)	0.15 (0.02, 1.21)			
**Education**
Primary School	Ref	0.066	Ref	0.262
High School	2.06 (0.23, 18.6)		1.76 (0.18, 17.5)	
University	5.14 (0.61, 43.0)		3.83 (0.38, 38.5)	
Family Status: without children vs. with children	2.21 (0.76, 6.38)	0.144	1.81 (0.50, 6.54)	0.368
**Stage of breast cancer**
I/II (early stage)	0.44 (0.15, 1.31)	0.05	0.42 (0.12, 1.47)	0.369
III/IV (late stage)	0.27 (0.09, 0.87)		0.65 (0.18, 2.38)	
Did not know			0.65 (0.18, 2.38)	
**Type of therapy**
Chemotherapy only	Ref	0.252		
Chemotherapy not used	2.87 (0.83, 9.99)			
Combination of chemotherapy and others	1.74 (0.62, 4.86)			
Knowing what oncofertility was: Yes vs. No	4.47 (1.87, 10.7)	0.001	3.73 (1.36, 10.2)	0.011
Knowledge of oncofertility (for each 1-point increase)	1.53 (1.09, 2.14)	0.014	1.69 (1.14, 2.51)	0.009

*OR, odds ratio; 95% CI, 95% confidence interval; AOR, adjusted odds ratio*.

a *Six patients reported “other” for their education and were excluded from the analysis*.

b *Only variables with p < 0.2 in the univariate logistic regression models were entered into the multiple logistic regression model*.

## Discussion

Our study revealed that slightly less than two-thirds of academic Chinese medical and surgical oncologists knew what the term oncofertility meant, though more than three quarters discussed at least some type of fertility preservation option. The majority of responding oncologists felt positively regarding offering these services, extended psychological support/counseling to their patients and encouraged partners to be part of the discussion. This contrasts with BCPs who were significantly less likely to know and discuss oncofertility with their oncologist and be offered/extended support services. Providers and BCPs' “knowing what oncofertility was” and level of knowledge were significantly associated with discussing fertility preservation options.

Since ASCO and ASRM published clinical practice guidelines in 2006 and with updates in 2012 adding oocyte cryopreservation as a standard practice for adults and children (11, 16, 34), there has been a world-wide increase in awareness for oncofertility referrals. This appears to be particular to BCPs who seemingly prefer to receive fertility-related information from reproductive endocrinology infertility specialists (35). However, fertility preservation remains one of the most under-prescribed and least implemented services in patients with cancer (35). This is particularly apparent for female fertility preservation, which is significantly more complex than male fertility preservation given the time required for ovarian stimulation and oocyte retrieval. In fact, data suggest that males are five times more likely to make arrangements for fertility preservation (36). Recently, oncofertility models of care including the EUropean REcommendations for female FERtility preservation (EU-REFER), which was a joint collaboration between oncologists and fertility specialists, has attempted to provide clinicians, and particularly oncologists, with a comprehensive standard reference when dealing with female cancer patients (37). Recommendations have included effective communication by oncologists, decision aids, age-appropriate care, referral pathways, documentation, training, supportive care during treatment, reproductive care after cancer treatment, psychosocial support, and ethical practice. Working as part of a multidisciplinary team, it is the hope that it will ensure that patients are less likely to miss out on receiving time-critical fertility information, which is potentially crucial to their chances of having children.

Despite the increasing awareness for oncofertility referrals, numerous previous qualitative studies have reported low actual rates of referrals (38–43). Several recent large population-based retrospective studies suggest that while referral rates for fertility assessment and treatment have increased not only for reproductive-aged cancer patients, pre-adolescent and adolescent patients, the overall referral and utilization of these services still remains remarkably low. This ranges from 4% for BCP [(35) and 1.7–3% in those with any cancer diagnosis in reproductive-aged women in the United States (44)]. This contrasts with European data which reveal slightly higher rates of referral (9%), though this still falls far from desired international guidelines (41, 45). Moreover, referrals have been inversely correlated with patient demographics, prognosis, advancing reproductive age, prior parity, and advanced disease. Patients' lack of awareness of oncofertility services together with the time pressures and conflicting priorities of physicians, reimbursement, and collaborative multi-disciplinary approaches continue to hinder adequate oncologist-patient fertility discussions and timely referrals appears to be a world-wide phenomenon (30, 37).

In China, models of care, referred to as multidisciplinary team (MDT) models, are slowly gaining traction and becoming increasingly prominent over the past 2 decades (46). Given the country's ever-increasing health care demands, imbalanced medical resource distribution, inadequate health insurance, and unsatisfactory implementation of disease management guidelines, it is clear that individual professional or discipline knowledge no longer is sufficient to cope with complicated medical conditions. Thus, the use of multidisciplinary collaborative guidance documents, such as systemic specifications of disease diagnosis and treatment, consultation systems, and protocols related to multi- disciplinary comprehensive management for difficult and severe diseases including cancer or critical diseases are being implemented (46). The goal is to diagnosis as soon as possible and treat patients in a timely and effective manner. With MDT's becoming more in-bread in the minds of physician leaders, models like EU-REFER model of care may serve as a template to enhance the awareness, create stream-line processes, and increase the utilization of fertility sparing options among health care professionals and AYA cancer patients (47–51). Since the inception of our current study, five Shanghai ART programs have since become part of the OC-NPC, though much work remains to be done. It is also crucial to educate physicians and patients about the requirements to enjoy oncofertility services such as the insurance coverage and the fact that—in contrast to elective fertility services—the patient does not have to be married.

Several limitations of this study should be acknowledged. First, the cross-sectional nature of this study precludes the ability to make conclusions on causation. For example, although more knowledge of oncofertility could be associated with a greater likelihood of discussing oncofertility with oncologists, BCPs who received discussion of oncofertility from their oncologist may be more likely to know what oncofertility was. Second, the relatively small sample size of oncologists and BCP respondents reduced statistical power to examine predictors of oncofertility knowledge and discussion. Third, since oncologists and BCPs were recruited from academic hospitals in Shanghai, the generalizability of our findings may be limited. Nevertheless, even in academic hospitals in Shanghai where medical education is relatively advanced in China, we found low referral rates for oncofertility among oncologists, as well as a large gap in the knowledge and service of oncofertility between oncologists and BCPs, which is concerning. In spite of these limitations, this study is valuable in providing support for continued study in this area. It provides useful insights into the knowledge gap and ineffective communication and comprehension that exists between Chinese oncologists and BCPs. Continued education and raised awareness are needed to optimize utilization of oncofertility services in China.

## Conclusions

In conclusion, this study reveals that most of the surveyed Chinese medical and surgical oncologists have a positive attitude toward oncofertility services; however, a lack of fertility preservation knowledge for both health-care providers and patients exists, which may hinder patient referrals. These findings emphasize the need for the standardization of oncofertility education and training as well as the need for a rapid and effective navigation mechanism between oncologists, cancer patients, and reproductive health specialists.

## Data Availability Statement

The original contributions presented in the study are included in the article/Supplementary Material, further inquiries can be directed to the corresponding author/s.

## Author Contributions

EB, ZX, SL, and HZ contributed to the study design, recruitment-analysis, and manuscript writing. JZ and RL contributed to manuscript writing (figures/tables) and study's statistical analysis. YS and YL contributed to the study recruitment practices and manuscript review. LA contributed to the study design, analysis, and manuscript writing. All authors contributed to the article and approved the submitted version.

## Funding

EB was supported by Krebsliga Schweiz, BIL KFS 4261-08-2017.

## Conflict of Interest

The authors declare that the research was conducted in the absence of any commercial or financial relationships that could be construed as a potential conflict of interest.

## Publisher's Note

All claims expressed in this article are solely those of the authors and do not necessarily represent those of their affiliated organizations, or those of the publisher, the editors and the reviewers. Any product that may be evaluated in this article, or claim that may be made by its manufacturer, is not guaranteed or endorsed by the publisher.
