# Supplementary material for: Oncofertility Knowledge and Communication: Comparison Between Medical and Surgical Oncologists and Breast Cancer Patients in Academic Chinese Centers
Source: Front Surg. 2021 Sep 7;8:681614. doi: 10.3389/fsurg.2021.681614 (PMC8453209; doi:10.3389/fsurg.2021.681614)
Supplement: Supplementary file 1 [file Data_Sheet_1.docx]

Appendix A

***China Oncofertility Survey***

***HCPs***

**Demographics and type of clinical practice**

1. What is your age?
   1. Xxx
2. You have been in practice how many years?
   1.  <5
   2.  5-10
   3.  10-20
   4.  >20
3. Please select your specialty:
   1. Medical oncologist – senior
   2. Medical oncologist-junior
   3. Surgical oncologist – senior
   4. Surgical oncologist-junior
   5. Gynecologist-senior
   6. Gynecologist-junior

**Oncofertility knowledge**

1. Do you know what oncofertility is?
   1.  Yes
   2.  No
2. Is male oncofertility treatment covered by insurance?
   1. Yes
   2. No
   3. I do not know
3. Is female oncofertility treatment covered by insurance?
   1. Yes
   2. No
   3. I do not know
4. Does a patient have to be married in order to undergo oncofertility?
   1. Yes
   2. No
   3. I do not know
5. What do you think is the cost of semen cryopreservation?
   1.  200 RMB
   2.  2000 RMB
   3.  10000 RMB
6. What do you think is the cost of oocyte/embryo cryopreservation?
   1.  2000 RMB
   2.  20000 RMB
   3.  50000 RMB
7. Oncofertility allows patients to access their gametes at any point of time in the future - correct?
   1. Yes
   2. No
   3. I do not know
8. How would you assess sperm function prior to cancer treatment?
   1.  Semen Analysis
   2.  Testicular Biopsy
   3.  I would not
   4.  I do not know
9. How would you assess ovarian reserve prior to cancer treatment?
   1.  FSH
   2.  AMH
   3.  I would not
   4.  I do not know
10. Do you know about oncofertility options from continuing medical education
    1.  Yes
    2.  No
11. Do you know about oncofertility options from media (journals, TV, internet etc.)?
    1.  Yes
    2.  No
12. How did you first learn about oncofertility?
    1. Hospital
    2. Congresses/conferences
    3. University
    4. Others (free text)

**Attitudes-perceptions**

1. How do you feel about offering fertility preservation options to cancer patients?
   1. Positive
   2. Negative
   3. I do not know
2. Why do you think are the reasons why adolescent-reproductive age cancer patients are not undergoing gamete cryopreservation?
   1.  own wish
   2.  finances
   3.  unawareness of option
   4.  cultural barriers to fertility preservation
   5.  I do not know

**Discussion**

1. With how many patients in the past year have you discussed sperm banking?
   1.  <10
   2.  10-25
   3.  25-50
   4.  >50
2. Do you discuss oncofertility with your reproductive-age patients?
   1.  Yes
   2.  No
3. Do you discuss oncofertility with your pre-adolescent age patients?
   1.  Yes
   2.  No
4. Do you discuss oncofertility with your adolescent age patients?
   1.  Yes
   2.  No
5. If yes, do you initiate the topic of oncofertility options?
   1.  Yes
   2.  No
6. If no, do you wait for the patient to initiate the topic of oncofertility options?
   1.  Yes
   2.  No
7. What oncofertility options did you discuss with your female patients?
   1.  Egg Banking
   2.  Embryo Banking
   3.  Ovarian Tissue Banking
   4.  GnRH-a Therapy
   5.  None
8. Which oncofertility options did you ever discuss with the male partners?
   1.  Sperm Banking
   2.  Testicular Biopsy
   3.  Testicle Freezing
   4.  None
9. Do your offer psychological support to your cancer patients?
   1.  Yes
   2.  No

**Utilization**

1. With how many patients in the past year have you discussed sperm banking?
   1.  <10
   2.  10-25
   3.  25-50
   4.  >50
2. How many patients in the past year have done sperm banking?
   1.  <10
   2.  10-25
   3.  25-50
   4.  >50
3. With how many patients in the past year have you discussed oocyte/embryo banking?
   1.  <10
   2.  10-25
   3.  25-50
   4.  >50
   5.  I do not know
4. How many patients in the past year have done oocyte/embryo banking?
   1.  <10
   2.  10-25
   3.  25-50
   4.  >50
   5.  I do not know

Appendix B

***China Oncofertility Survey***

***Breast Cancer patients***

**Demographics**

1. What is your age?
   1. Xxx
2. What is your highest education?
   1. Primary school
   2. High school
   3. University
3. Where do you live?
   1. Shanghai
   2. Outside of Shanghai in a city≥ 1ml inhabitants
   3. Outside of Shanghai in a city≤ 1ml inhabitants
   4. Rural area
4. What is your marital/family status?
   1. Married with children
   2. Married without children
   3. Single
   4. Divorced with children
   5. Divorced without children
   6. I have a boyfriend and I have children
   7. I have a boyfriend and I have no children
   8. I am engaged and I have children
   9. I am engaged and I have no children
5. What is/was your stage of breast cancer at diagnosis?
   1. I (a or b or c)
   2. II (a or b)
   3. III (a or b or c)
   4. IV (palliative)
   5. Paget disease
6. What kind of therapy did you or will you receive? (multiple choice)
   1. Chemotherapy
   2. Radiotherapy
   3. Endocrine therapy
   4. Immunotherapy
   5. Palliative therapy

**Knowledge**

1. Have you ever heard of the term oncofertility?
   1.  Yes
   2.  No
2. Is the oncofertility treatment covered by your insurance?
   1. Yes
   2. No
   3. I do not know
3. Do you know if you have to be married in order to undergo oncofertility?
   1. Yes
   2. No
   3. I do not know
4. Oncofertility allows patients to access their gametes (eggs or sperm) at any point of time in the future – right?
   1. Yes
   2. No
   3. I do not know
5. Did you know about oncofertility options from media (journals, TV, internet etc.)?
   1.  Yes
   2.  No
6. What do you think is the cost of oocyte/embryo cryopreservation?
   1.  2000 RMB
   2.  20000 RMB
   3.  50000 RMB

**Discussion**

1. Did you discuss oncofertility with your doctor/reproductive-age patients?
   1.  Yes
   2.  No
2. If yes, who did start the topic of oncofertility options?
   1. The doctor-nurse
   2. Me as the patient
3. What oncofertility options did you discuss?
   1.  Egg Banking
   2.  Embryo Banking
   3.  Ovarian Tissue Banking
   4.  GnRH-a Therapy
   5.  All of the above
   6. None
4. If not with your cancer doctor/nurse, who else did you discuss oncofertility with?
   1.  Other doctors
   2.  Nurses
   3.  Family/friends
   4.  Other (free comment space)
   5.  No one
5. Was your partner involved in the discussions about oncofertility?
   1.  Yes
   2.  No
   3. I don’t have one
6. Were you told how you could assess ovarian reserve prior to and after cancer treatment?
   1.  FSH
   2.  AMH
   3.  I would not
   4.  I do not know
7. Were you offered psychological support?
   1.  Yes
   2.  No
8. Did you get psychological support?
   1.  Yes
   2.  No
